# Biochar loaded with cobalt ferrate activated persulfate to degrade naphthalene

**DOI:** 10.1039/d2ra08120b

**Published:** 2023-02-10

**Authors:** Shuaijie Gu, Jingying Cui, Fangqin Liu, Jinyang Chen

**Affiliations:** a School of Environmental and Chemical Engineering, Shanghai University 99 Shangda Road Shanghai 200444 PR China gushuaijie@shu.edu.cn

## Abstract

Considering the simple preparation of biochar and the excellent activation performance of cobalt ferrate material, a biochar supported cobalt ferrate composite was synthesized by a solvothermal method. The material was used to activate persulfate (PS) to degrade naphthalene (NAP) in water. The structure and morphology characterization showed that the composite (CoFe_2_O_4_-BC) was successfully prepared. Under the conditions of 0.25 g L^−1^ CoFe_2_O_4_-BC and 1 mM PS, 90.6% NAP (the initial concentration was 0.1 mM) was degraded after 30 minutes. The degradation kinetics of NAP followed the pseudo-first-order kinetic model with a rate constant of 0.0645 min^−1^. With the increase of the dosage of activator and PS, the removal rate of NAP could be increased to 99.5%. The coexistence of anions and humic acids inhibited the removal of NAP. The acid environment promoted the removal of NAP while the alkaline environment inhibited it. After four cycles of CoFe_2_O_4_-BC material, the removal rate of NAP decreased from 90.6% to 79.4%. The removal of TOC was about 45% after each cycle. After the first cycle, the concentration of leached cobalt ion and leached iron ion was about 310 μg L^−1^ and 30 μg L^−1^ respectively. The free radical quenching experiments showed that SO_4_^−^˙ and OH˙ were the main causes of NAP removal, and the possible degradation path of NAP was elucidated by DFT calculation.

## Abstract 2

Considering the simple preparation of biochar and the excellent activation performance of cobalt ferrate material, a biochar supported cobalt ferrate composite was synthesized by a solvothermal method.

## Graphical abstract

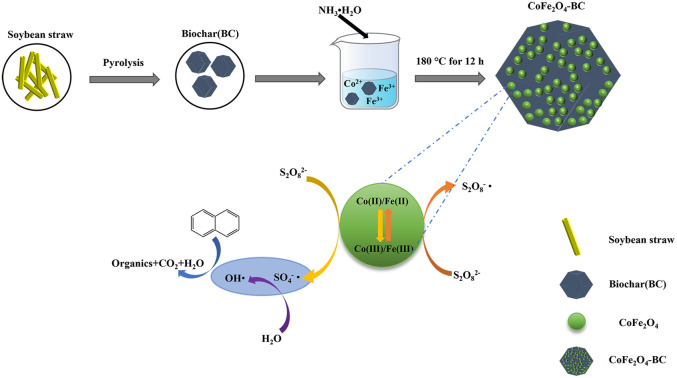

## 1. Introduction

Polycyclic aromatic hydrocarbons (PAHs) have mostly been identified as carcinogenic, teratogenic, mutagenic^1,2^ and biologically cumulative,^3^ and have been found in water,^4,5^ air,^6^ soil,^7,8^ and sediment.^9^ They are a class of non-polar, semi-volatile organic compounds, whose hydrophobicity increases with the increase of the number of aromatic rings.^10–12^ Compared with other media, PAHs are mainly adsorbed on soil and sediment,^13,14^ and in the environment are mainly from industrial and automobile exhaust emissions.^15,16^

As for the dispose of PAHs pollution, researchers have studied many remediation methods, including physical,^17^ chemical^18–20^ and biological method.^21^ Among them, advanced oxidation process (AOPs) of chemical restoration method has attracted much attention for its high efficiency and green advantages, including photocatalytic method,^22^ electrocatalytic method (EC),^23^ Fenton method,^24^ persulfate (PS) method^25^ and peroxymonosulfate (PMS) method.^26^ AOPs has been widely used to degrade refractory organic pollutants because of its ability to generate highly active substances, such as hydroxyl radical (OH˙), sulfate radical (SO_4_^−^˙) and superoxide anion radical (O_2_^−^˙), which can degrade organic pollutants into carbon dioxide (CO_2_), water (H_2_O) and small organic compounds.

The advantages of PS/PMS oxidation are that SO_4_^−^˙ has a higher REDOX potential (2.5–3.1 V) than OH˙ (1.8–2.7 V),^27^ is safer and has a longer half-life (30–40 μs).^28^ In addition, SO_4_^−^˙ can effectively react with target contaminants in a wide pH range (2–9).^29,30^ Although PS/PMS are excellent SO_4_^−^˙ precursors, they are usually not feasible for persulfate to directly react with most pollutants. For more efficient degradation of target pollutants, the participation of activators is required. Various activation methods have been proposed, including the use of thermal,^31^ ultraviolet,^32^ ultrasonic,^33^ electrochemical^34^ and transition metal material.^35^ Among these activation methods, transition metal has the characteristics of simple operation, high activation efficiency, low cost and wide applicability. Transition metal ions were the first discovered activators,^36^ in addition to iron and its oxides,^35,37^ Mn oxides^38^ and Co oxides.^39^ Bimetallic activators have higher chemical stability (less leached ions), higher REDOX activity and better activation activity.^40–42^ Bimetallic oxides such as CoMn_2_O_4_,^43^ CuFeO_2_,^44^ NiFe_2_O_4_,^45^ CoFe_2_O_4_,^26^ and MnFe_2_O_4_ (ref. 46) have also been studied as activators. However, as heterogeneous activators, they still have the problem of easy aggregation.^26,47^

In order to solve the problem of aggregation, the studies of loading the transition metal activator materials with zeolite,^47^ reduced graphene oxide (r-GO)^26^ and Biochar (BC),^48^ have been reported. Compared to other carriers, BC has the advantages of well-developed pores, low cost, simple preparation^49,50^ and abundant oxygen-containing functional groups,^49,51^ such as hydroxyl (–OH) and carboxyl (–COOH). BC not only has been used as an effective and low-cost adsorbent for the removal of organic pollutant^52^ and heavy metal,^53^ but also can activate Fenton system^50^ and persulfate system.^54^ Furthermore, with biochar as the carrier, the activation performance of the activator can increase 1.5–3 times.^48,55,56^ In the system, biochar mainly acts as an electron transport medium,^48^ and effectively inhibits the aggregation of activators, which increases the specific surface area of activators.^56^ Therefore, using carbonaceous material as the carrier can not only effectively improve the activation of activator, but also reduce the cost. Fe and Co elements have strong ability to activate persulfate to generate free radical.^36^ Cobalt ferrite, one of the magnetic spinel ferrites, has attracted great interest because of its large specific surface area, good chemical stability and low metal ion leaching rate.^40,42^ Therefore, with biochar supported cobalt ferrite composites to degradation of naphthalene is very important.

In the study, biochar (BC) was produced by pyrolysis of crop straws, and biochar supported cobalt ferrate composite (CoFe_2_O_4_-BC) was prepared by solvothermal method. In the CoFe_2_O_4_-BC activated persulfate system, the degradation kinetics of naphthalene (NAP), influence of various factors, activation mechanism, possible degradation path of NAP, mineralization rate and recyclability of materials were studied.

## 2. Materials and methods

### 2.1. Materials

Cobalt nitrate hexahydrate (Co(NO_3_)_2_·6H_2_O), iron nitrate 9 hydrate (Fe(NO_3_)_3_·9H_2_O), sodium bicarbonate (NaHCO_3_), sodium chloride (NaCl) and sulfuric acid (H_2_SO_4_) were purchased from China National Pharmaceutical Group Co., Ltd. Naphthalene (NAP), acetone, *tert*-butanol (TBA), humic acid (HA), sodium hydroxide (NaOH) and potassium persulfate (K_2_S_2_O_8_) were purchased from Shanghai Maclin Biochemical Technology Co., LTD. Ammonia (NH_3_·H_2_O) and *N*,*N* dimethylformamide (DMF) were purchased from China Discovery Platform (Tansoole). All reagents mentioned above are analytically pure. Methanol (MeOH, HPLC). Deionized water (resistivity ≥18.2 MΩ). Soybean straw purchased from Lianyungang, Jiangsu, China.

### 2.2. Preparation of BC, CoFe_2_O_4_ and CoFe_2_O_4_-BC

Biochar (BC) was prepared by pyrolysis method:^49^ soybean straw was crushed first. The powder was washed and dried after passing the 2 mm screen surface. Then the soybean straw powder is moved into the vacuum tube furnace. Under the condition that the heating rate is 5°C min^−1^ and the shielding gas is argon, the temperature rises to 450 °C for 2 h. After cooling in the tubular furnace, the biochar was ground and passed through a 100-mesh screen surface (aperture 0.15 mm) and stored in a brown sealed bottle.

CoFe_2_O_4_ and CoFe_2_O_4_-BC were prepared by solvothermal method, referring to previous studies:^56,57^ firstly, biochar (about 235 mg) was added to 30 mL deionized water and subjected to 1 h ultrasonic treatment to prepare a uniform biochar dispersion. Meanwhile, 1 mmol Co(NO_3_)_2_·6H_2_O and 2 mmol Fe(NO_3_)_3_·9H_2_O were dissolved in 5 mL deionized water and 30 mL DMF, and the mixture was magnetically stirred at room temperature for 30 minutes. The resulting mixture is then added to the biochar dispersion by drops under magnetic agitation. After 30 minutes, 5 mL ammonia solution was injected into the above mixture and stirred for another hour. Transfer the final mixture to a reaction kettle (100 mL PTFE lined) and heat at 180 °C for 12 h. Cool naturally to room temperature. Washing (with deionized water and ethanol) and centrifugation until the pH of the wash solution was 7. Finally, dry at 60 °C for 24 hours, gently grind into powder and pass a 100-mesh screen surface. Through these steps, CoFe_2_O_4_-BC powder can be obtained. CoFe_2_O_4_ powder was prepared in the same way, but without the addition of BC.

### 2.3. Characterizations

X-Ray diffractometer (D/MAX2200V PC (3 KW) model in Japan) was used to determine the XRD patterns of the samples. SEM images and EDS tests of the samples were taken with ZEISS GeminiSEM 300 scanning electron microscope. The accelerated voltage during morphology shooting was 3 kV, the accelerated voltage during energy spectrum mapping was 15 kV and the detector was SE2 secondary electron detector. XPS was determined using Thermo Scientific K-Alpha. The spot size is 400 μm, the working voltage is 12 kV, and the filament current is 6 mA. The full-spectrum scanning pass energy is 150 eV, and the step size is 1 eV. Narrow-spectrum scanning has a pass energy of 50 eV and a step size of 0.1 eV. Nitrogen adsorption and desorption of samples were tested by using a 3-station automatic surface area analyzer (Quantachrome Autos orb IQ3) under 77 k liquid nitrogen conditions. Hysteresis loops of sample was measured by instrument (LakeShore 7404).

### 2.4. Degradation of naphthalene and analytical methods

Preparation of naphthalene solution: firstly, 128 mg of NAP particles were dissolved in 10 mL of acetone (cosolvent) to make a reserve solution, then 1 mL of the reserve solution was added to 1000 mL deionized water (the ratio of acetone to water was 1 : 1000), finally, magnetic stirring (700 rpm) for 2.5 hours to obtain 1 mM of naphthalene solution.

Add 100 mL naphthalene solution to 250 mL beaker, then add oxidant and activator. Using automatic mechanical stirring, the rotor speed is 200 rpm. The rotor is made of plastic, 4 cm in diameter and about 1.0 cm from the bottom of the beaker. Unless otherwise stated, the sampling time is usually set as 5, 10, 15, 20, 30 min, the dosage of activator is 0.25 g L^−1^, the concentration of PS is 1 mM, the initial pH of the solution is 7, and the reaction temperature is room temperature (25 °C).

Sampling procedure: 0.5 mL methanol was added to the sample bottle, then 1.0 mL solution to be analyzed was added through 0.22 μm organic filtration membrane, and the measurement was completed within 24 h (methanol was used to stop the reaction).

The concentration of naphthalene was determined by a high-performance liquid chromatograph (Agilent 1260) equipped with an EC-C18 column (4.6 mm × 150 mm, 5 μm). The UV detection wavelength was 254 nm, the mobile phase was a mixture of ultra-pure water and methanol (HPLC grade) (25 : 75 (v/v)), the column temperature was 25 °C and the sample size was 10 μL. TOC was determined by total organic carbon analyzer (Multi NC 3100, Analytik Jena). Metal ion content was determined by inductively coupled plasma (Analytikjena PQ 9000).

## 3. Results and discussion

### 3.1. Characterizations

**Fig. 1:**
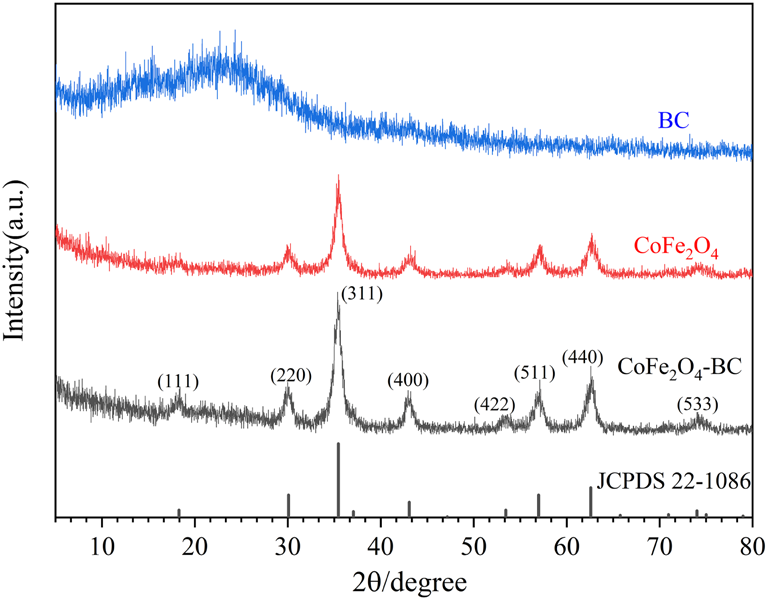
XRD patterns of BC, CoFe_2_O_4_ and CoFe_2_O_4_-BC.

XRD patterns is given in Fig. 1. The wide peaks of 20° to 25° in the XRD pattern of BC represent disordered graphite planes, indicating that BC is not completely graphitized.^49,58^ The XRD pattern of the prepared CoFe_2_O_4_ and JCPDS no. 22-1086 card can be accurately matched. The characteristic diffraction peaks are 18.3°, 30.1°, 35.4°, 43.1°, 53.4°, 57.0°, 62.6° and 74.0°, corresponding to crystal planes (111), (220), (311), (400), (422), (511), (440) and (533) respectively. The relatively sharp and narrow diffraction peaks indicate that the sample crystallizes well, and the highly crystalline properties of the CoFe_2_O_4_ sample do not change after forming the composite with BC.

**Fig. 2:**
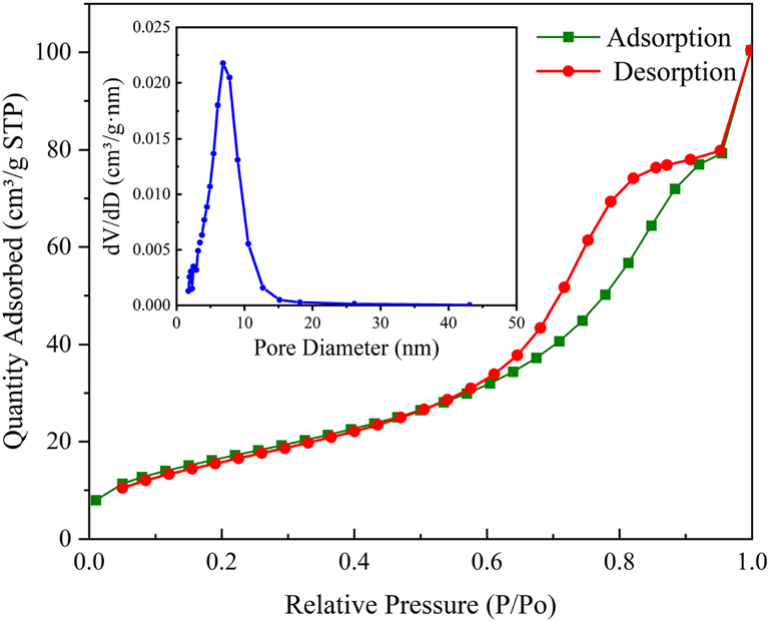
N_2_ adsorption–desorption isotherm of CoFe_2_O_4_-BC (illustration: pore size distribution curve).

As shown in Fig. 2, the N_2_ adsorption–desorption isotherm of CoFe_2_O_4_-BC represents a typical type IV isotherm, corresponding to the characteristics of the porous structure. The pore size distribution curve indicates that the pore size in the mesoporous area is mainly distributed between 2–12 nm, and the average pore size is 8.19 nm. The specific surface area was calculated by BET method and was 62.85 m^2^ g^−1^. The pore volume was calculated by BJH method and was 0.1563 cm^3^ g^−1^.

**Fig. 3:**
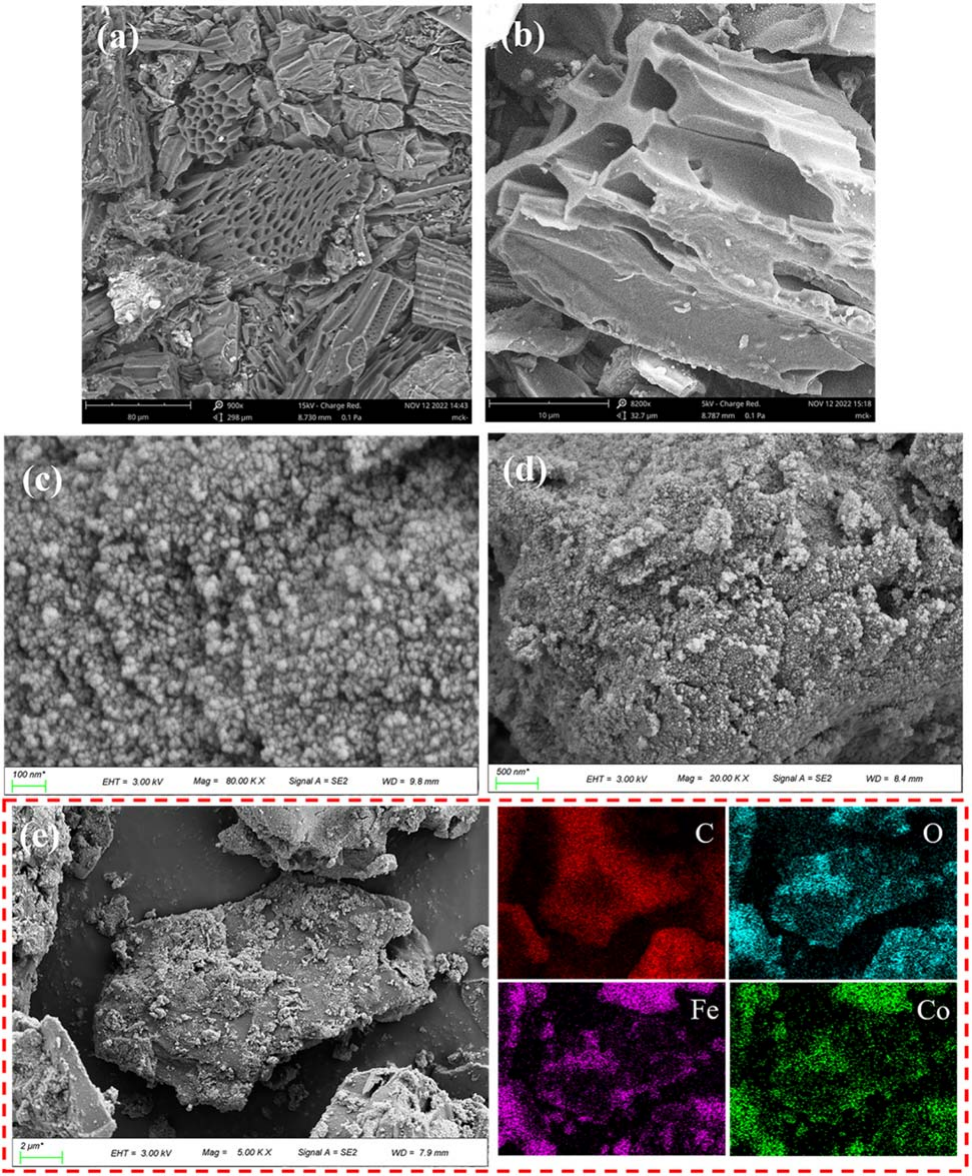
SEM image of BC (a and b); SEM (c and d), EDS elemental mappings (e) of CoFe_2_O_4_-BC.

SEM images were shown in Fig. 3a and b, BC has a lamellar, rough, irregular and porous structure, which is a typical structure of biochar.^55^ Furthermore, it can obtain from Fig. 3e that the elements C, O, Fe and Co are evenly distributed in the material, and the atomic number ratio of the four elements of C, O, Fe and Co is 40 : 7 : 2 : 1. According to Fig. 3c and d, CoFe_2_O_4_-BC material has a bulk structure, CoFe_2_O_4_ particles are well dispersed on the surface of BC, and the particle size of CoFe_2_O_4_ particles is about 20–50 nm.

**Fig. 4:**
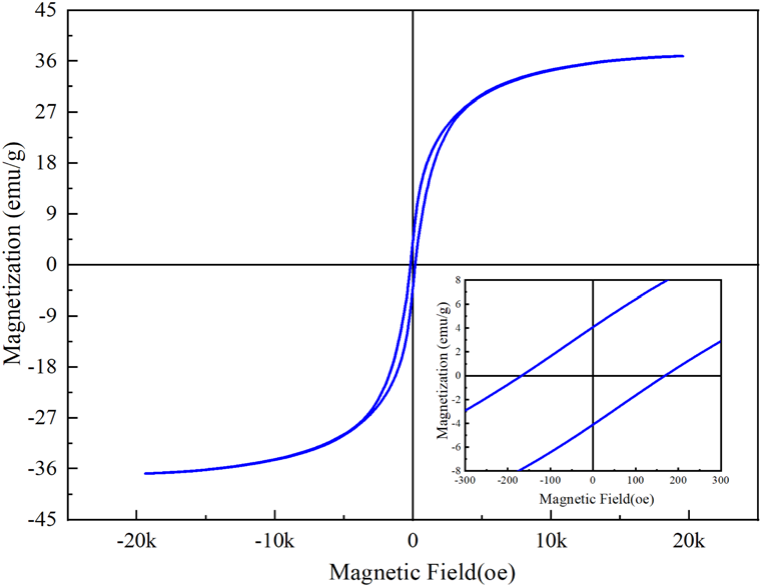
Hysteresis curve of CoFe_2_O_4_-BC.

Fig. 4 below shows the measured magnetic results of sample CoFe_2_O_4_-BC. It can be seen from this hysteresis curve that the saturation magnetization (Ms), remanence (Mr) and coercivity (Hc) of CoFe_2_O_4_-BC are 36.8 emu g^−1^, 4.07 emu g^−1^ and 171 Oe, which are closely related to the magnetic properties of the material.

**Fig. 5:**
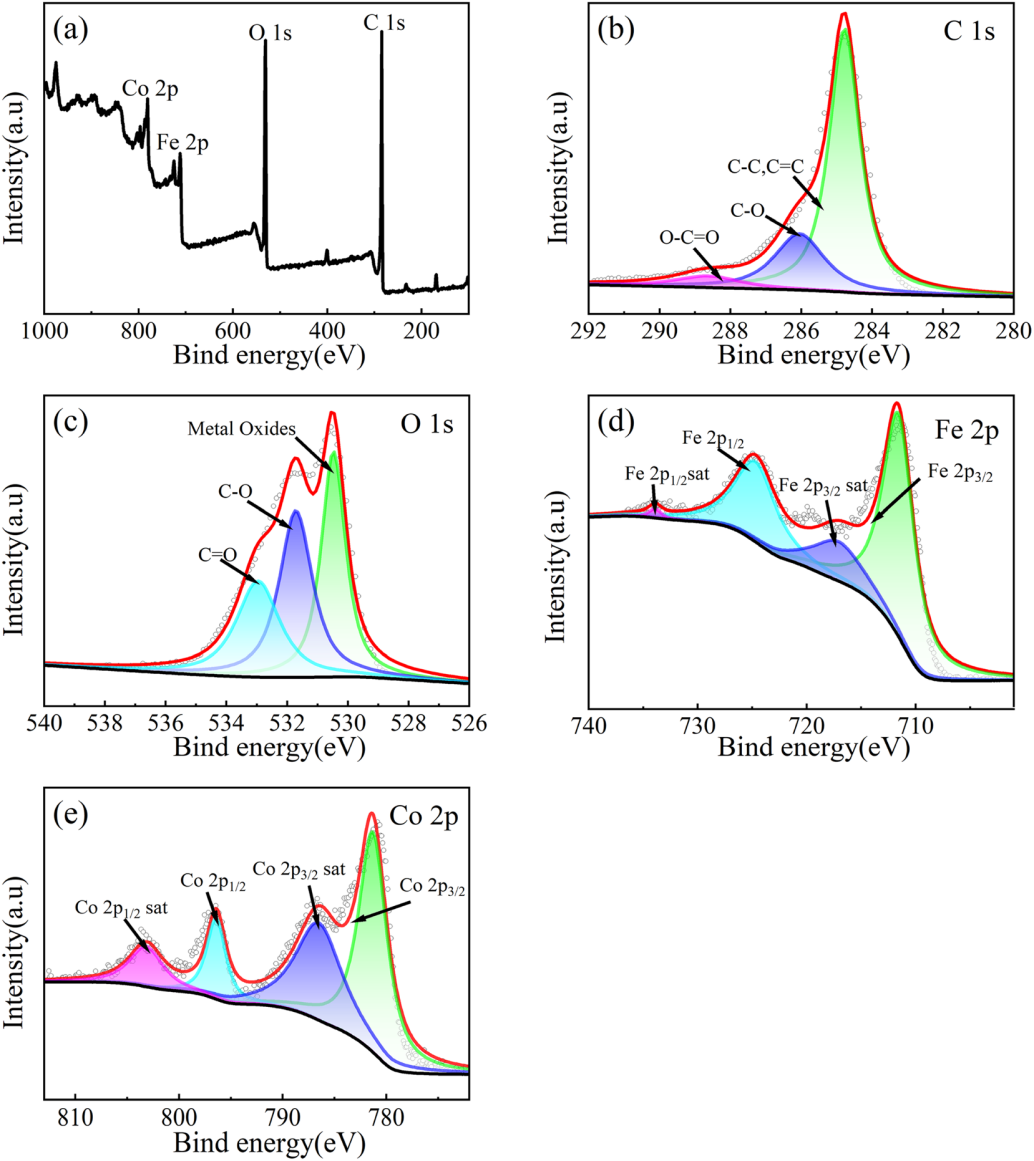
XPS spectrum of CoFe_2_O_4_-BC: full spectrum (a), C 1s (b), O 1s (c), Fe 2p (d) and Co 2p (e).

XPS is used to analyze the chemical states of elements and it is shown in Fig. 5. According to Fig. 5b, the C 1s XPS spectrum can be fitted to three peaks, corresponding to the C–C (284.78 eV), C–O (286.05 eV) and O–C=O (288.63 eV) bonds.^59^ As shown in Fig. 5c, the O 1s XPS spectrum can be fitted to three peaks, corresponding to the Co–O/Fe–O (530.47 eV), C–O (531.17 eV) and C=O (532.96 eV) bonds.^60^ As shown in Fig. 5d, the Fe 2p XPS spectrum can be fitted to four peaks corresponding to Fe 2p_3/2_ spin orbit peak (711.75 eV), Fe 2p_1/2_ spin orbit peak (724.85 eV) and two satellite peaks of two spin orbits (717.7 eV, 733.9 eV), which is the main state of Fe^3+^. Therefore, it can be proved that the valence state of Fe is +3 valence.^60,61^ As shown in Fig. 4e, Co 2p XPS spectrum can be fitted to four peaks, corresponding to Co 2p_3/2_ spin orbit peak (781.3 eV), Co 2p_1/2_ spin orbit peak (796.4 eV) and two satellite peaks of two spin orbits (786.5 eV, 803.1 eV). The numerical difference of the spin orbital peak of 15.1 eV is a sign of Co^2+^, so it can be proved that the valence state of Co is +2 valence.^61^

### 3.2. Degradation kinetics of naphthalene by CoFe_2_O_4_-BC/PS

**Fig. 6:**
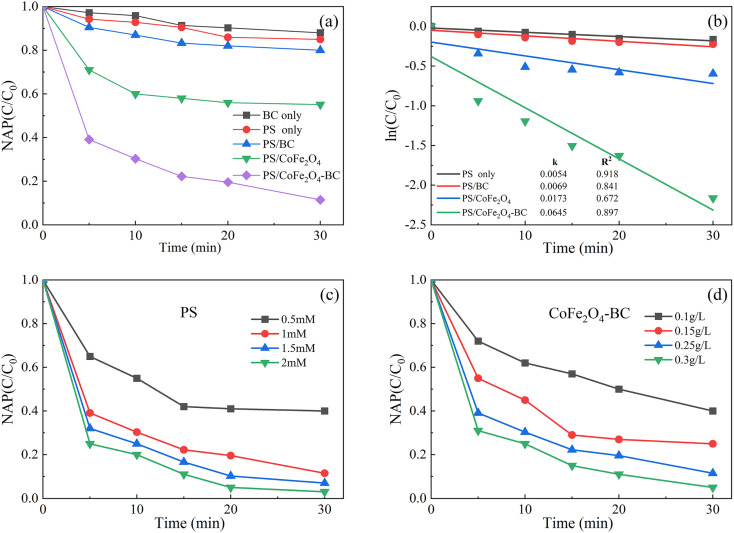
Residual NAP (a) and kinetics (b) in different systems; the effect of the initial concentration of PS (c) and the dosage of activator (d). ([NAP] = 0.1 mM, pH = 7, *T* = 25 °C).

As shown in Fig. 6a, after 30 min of reaction, BC could remove 12.0% NAP, indicating that biochar could absorb a certain amount of organic pollutant, which was the same as published studies.^55,62^ The removal rate of NAP in PS system was 15.1%, that in PS/BC system was 20.5%, and that in PS/CoFe_2_O_4_ system was 48.9%. The removal rate of NAP in PS/CoFe_2_O_4_-BC system was 90.6%, indicating that CoFe_2_O_4_-BC could activate PS well to remove NAP. In addition, 87.56% naphthalene was removed by photocatalyst after 140 minutes,^63^ and 90% was removed by biological method after 28 hours,^64^ which demonstrated the high efficiency of the persulfate/activator system.

As shown in Fig. 6b. The degradation kinetics of NAP was fitted by the eqn (1) (*C*_0_ is the initial concentration, *C* is the concentration at different times, *k* is the first-order rate constant (min^−1^), and *T* is the reaction time (min)). The degradation kinetics of NAP followed the pseudo-first-order model well.

1 ln(*C*/*C*_0_) = −*kT*

The corresponding rate constants of PS, PS/BC, PS/CoFe_2_O_4_ and PS/CoFe_2_O_4_-BC were 0.0054 min^−1^, 0.0069 min^−1^, 0.0173 min^−1^ and 0.0645 min^−1^, respectively.

As shown in Fig. 6c and d, under the condition that the dose of activator was 0.25 g L^−1^, the dose of PS increased from 0.5 mM to 2 mM, and the removal rate of NAP increased from 58% to 99.7%. When the dose of PS was 1 mM, the dose of activator increased from 0.1 g L^−1^ to 0.3 g L^−1^, and the removal rate of NAP increased from 60% to 99.5%. It is proved that the material can effectively activate persulfate to completely remove NAP under certain conditions.

### 3.3. Influence of coexisting anions, initial pH and HA

**Fig. 7:**
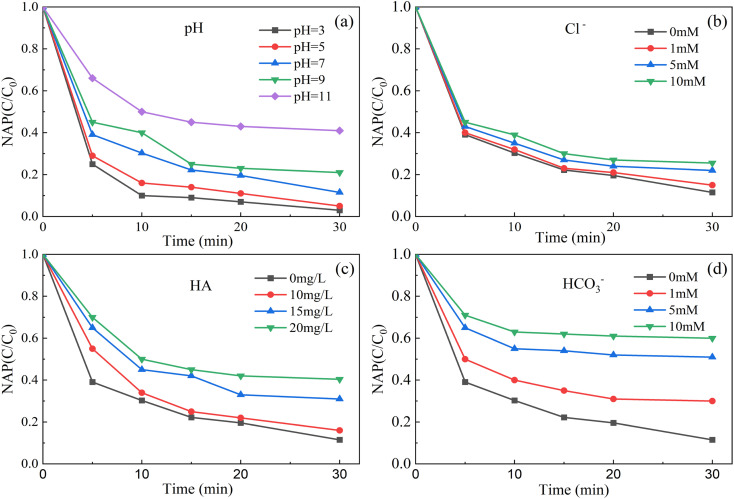
Effect of initial pH (a), Cl^−^ (b), HA (c) and HCO_3_^−^ (d) on removal of NAP. ([NAP] = 0.1 mM, [CoFe_2_O_4_-BC] = 0.25 g L^−1^, [PS] = 1.0 mM, pH = 7, *T* = 25 °C).

As shown in Fig. 7a, when the initial pH of the solution was 3, the degradation rate of NAP was 99.0%. When the initial pH of the solution was 11, the degradation rate of NAP was 59.1%. That is, the lower the pH value of solution, the higher the degradation efficiency of NAP, and the higher the pH value of solution, the lower the degradation rate of NAP. The main reasons are as follows.

Acidic conditions may accelerate the decomposition of persulfate to SO_4_^−^˙, resulting in increased degradation of target pollutants (eqn (2)). However, under alkaline conditions, SO_4_^−^˙ will be consumed (eqn (3)), resulting in reduced degradation of pollutants.^65^ Although OH˙ has oxidation capacity, SO_4_^−^˙ has a higher REDOX potential (2.5–3.1 V) and a longer half-life (30–40 μs)^27^ than OH˙ (1.8–2.7 V).^28^ Similar to the results of published literature,^62^ the degradation of atrazine by the nano-zero-valent iron-persulfate system at different pH conditions was also in this trend.

2 S_2_O_8_^2−^ + H^+^ → HS_2_O_8_^−^ → SO_4_^2−^ + SO_4_^−^˙+ H^+^

3 SO_4_^−^˙ + OH^−^ → SO_4_^2−^ + OH˙

As shown in Fig. 7b–d, in this experiment, the degradation rate of NAP decreased with the increase of the dosage of Cl^−^, HCO_3_^−^ and HA. The main reasons are as follows. Cl^−^ and HCO_3_^−^ are generally considered to inhibit the degradation of pollutants.^66,67^ They can lead to the conversion of powerful SO_4_^−^˙ and OH˙ into less reactive chlorine and carbonate radicals (eqn (4)–(8)).^42,68^ The OH˙ in the system can be converted from SO_4_^−^˙ (eqn (4)^65^).

4 SO_4_^−^˙ + H_2_O → OH˙ + SO_4_^2−^ + H^+^

5 Cl^−^ + SO_4_^−^˙ → SO_4_^2−^ + Cl˙

6 Cl^−^ + OH˙ → Cl˙ + OH^−^

7 HCO_3_^−^ + SO_4_^−^˙ → CO_3_^−^˙ + SO_4_^2−^ + H^+^

8 HCO_3_^−^ + OH˙ → CO_3_^−^˙ + H_2_O

HA is considered as a natural polyelectrolyte and organic compound with complex structure, containing carboxylate, methoxy, hydroxyl, phenolic, quinone and other functional groups.^69–71^ Because of the particularity of HA structure, the existence of HA enhances or inhibits the degradation of target pollutants by oxidation system. Fang *et al.*^72^ found that the circulation of quinone and semi-quinone free radicals in HA can break down PS to enhance the degradation of 2,4,4′-trichlorobenzene. Li *et al.*^73^ pointed out that reactive radicals can be consumed by HA, which results in reduced removal of indomethacin by the PS/Fe^2+^ system. Yang *et al.*^74^ showed that the addition of HA can reduce the degradation of tetracycline in UV/H_2_O_2_ system.

### 3.4. Radical quenching experiment

A variety of radical scavengers have been used to determine the role of radicals in the catalytic process. The secondary reaction ratio constants of common free radical scavenger are shown in Table 1. Methanol (MeOH) and ethanol (EtOH) are effective against both SO_4_^−^˙ and OH˙. *tert*-Butanol (TBA) is widely used as an OH˙ scavenger and is relatively insensitive to SO_4_^−^˙. In addition to SO_4_^−^˙ and OH˙, superoxide radicals (O_2_^−^˙)^55^ and singlet oxygen (^1^O_2_)^75^ have also been detected in the PS system. Chloroform (CF) and benzoquinone (BQ) have been reported as O_2_^−^˙ scavengers. Chemicals such as furfuryl alcohol (FFA) and sodium azide (NaN_3_) can be used as ^1^O_2_ scavengers.

**Table 1:** Rate constant of radicals for different scavengers

Scavengers	Rate constant (M^−1^ s^−1^)				
	OH˙	SO_4_^−^˙	O_2_^−^˙	^1^O_2_	Ref.
EtOH	(1.2–1.9) × 10^9^	(1.6–7.7) × 10^7^	<10 ^3^	3.8 × 10^3^	55, 76 and 77
MeOH	(1.2–2.8) × 10^9^	(1.6–7.7) × 10^7^	—	—	76 and 78
TBA	(3.8–7.6) × 10^8^	(4.0–9.1) × 10^5^	<10^3^	< 10^4^	55, 77 and 79
CF	—	—	(1.1–3.2) × 10^9^	—	77 and 80
BQ	1.2 × 10^9^	1.0 × 10^8^	(0.9–1.9) × 10^9^	6.6 × 10^7^	55 and 81
FFA	1.5 × 10^10^	—	—	1.2 × 10^8^	77 and 80
NaN_3_	1.2 × 10^10^	2.5 × 10^9^	—	1 × 10^9^	28 and 55

**Fig. 8:**
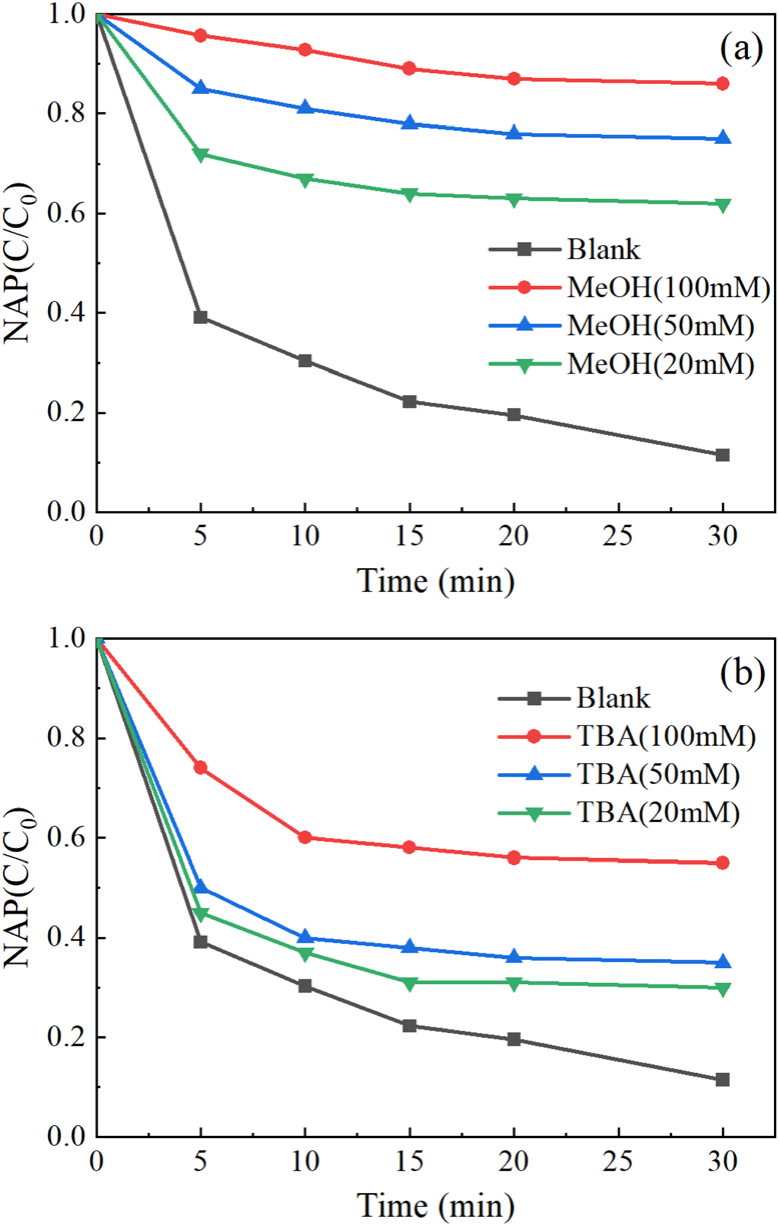
Free radical quenching experiments: MeOH (a), TBA (b). ([NAP] = 0.1 mM, [CoFe_2_O_4_-BC] = 0.25 g L^−1^, [PS] = 1.0 mM, pH = 7, *T* = 25 °C).

As shown in Fig. 8, the addition of excessive TBA moderately inhibited the removal of NAP, while the addition of excessive MeOH highly inhibited the removal of NAP. Therefore, SO_4_^−^˙ and OH˙ are the main reasons for the removal of NAP.

According to the published studies, the mechanism of CoFe_2_O_4_-BC activation of PS can be described by eqn (9)–(13) and (4) (eqn (9),^82^eqn (10),^82,83^eqn (11),^84^eqn (12),^85^eqn (13)^82^). These processes achieve Co(ii) regeneration: Co(ii) → Co(iii) → Co(ii), which is responsible for the effectiveness and stability of the activator.^86^ In addition, BC can not only support CoFe_2_O_4_ particles, but also acts as an electron transfer medium to accelerate these reaction processes.^48^

9 Co(ii) + S_2_O_8_^2−^→ Co(iii) + SO_4_^2−^ + SO_4_^−^˙

10 Fe(iii) + S_2_O_8_^2−^→ Fe(ii) + S_2_O_8_^−^˙

11 Fe(ii) + S_2_O_8_^2−^ → Fe(iii) + SO_4_^2−^ + SO_4_^−^˙

12 Co(iii) + Fe(ii) → Fe(iii) + Co(ii)

13 Co(iii) + S_2_O_8_^2−^ → Co(ii) + S_2_O_8_^−^˙

### 3.5. Exploration of activator stability, activator recyclability and mineralization rate

After each experiment, an external magnetic field (from a magnetic agitator) was used to aggregate the CoFe_2_O_4_-BC particles. Part of the upper solution was separated, and centrifuged to collect CoFe_2_O_4_-BC particles. Finally, wash with deionized water several times and dry for the next experiment. The stability of the material was judged by measuring the concentration of leached metal ions, and the mineralization rate of the system was calculated by measuring the total organic carbon content (TOC) of the solution before and after the reaction.

**Fig. 9:**
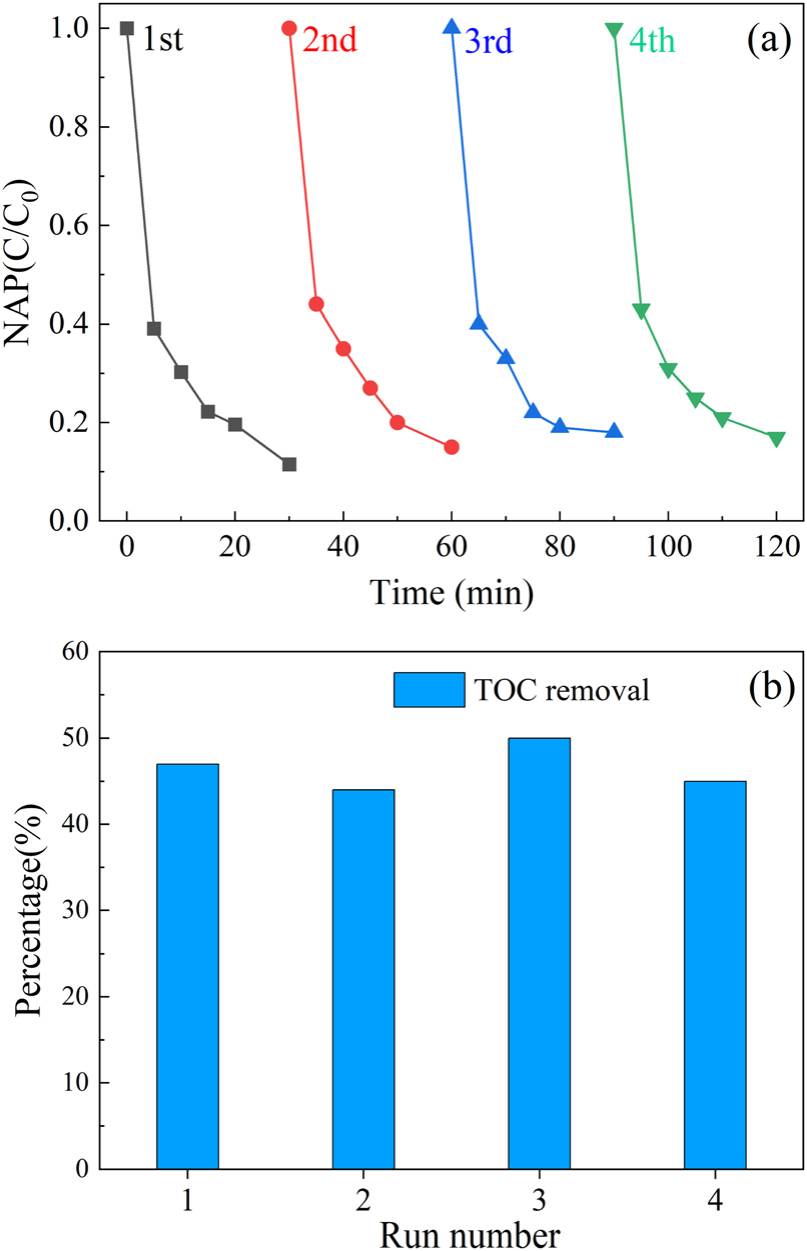
Recycling performance of activator (a); TOC removal (b) in each cycle. ([NAP] = 0.1 mM, [CoFe_2_O_4_-BC] = 0.25 g L^−1^, [PS] = 1.0 mM, pH = 7, *T* = 25 °C).

As shown in Fig. 9a, after four cycles of experiments, the removal rate of NAP decreased from 90.6% to 79.4%, and the performance decreased. As shown in Fig. 9b, after each cycle experiment, the removal rate of total organic carbon (TOC) in the solution was about 45%. Furthermore, after the first cycle, the concentration of leached cobalt ion was about 310 μg L^−1^ and the concentration of leached iron ion was about 30 μg L^−1^, which was far lower than the European Union limit standard (2.0 mg L^−1^)^87^ and proves that the composite material has strong stability.

### 3.6. Analyse the possible degradation pathways of NAP based on DFT theoretical calculation

Rapid advances in computational chemistry have made it possible to predict reaction sites for relatively large molecules. Fukui function calculations based on density functional theory (DFT) are common tools for predicting free radical selection reaction regions.

**Fig. 10:**
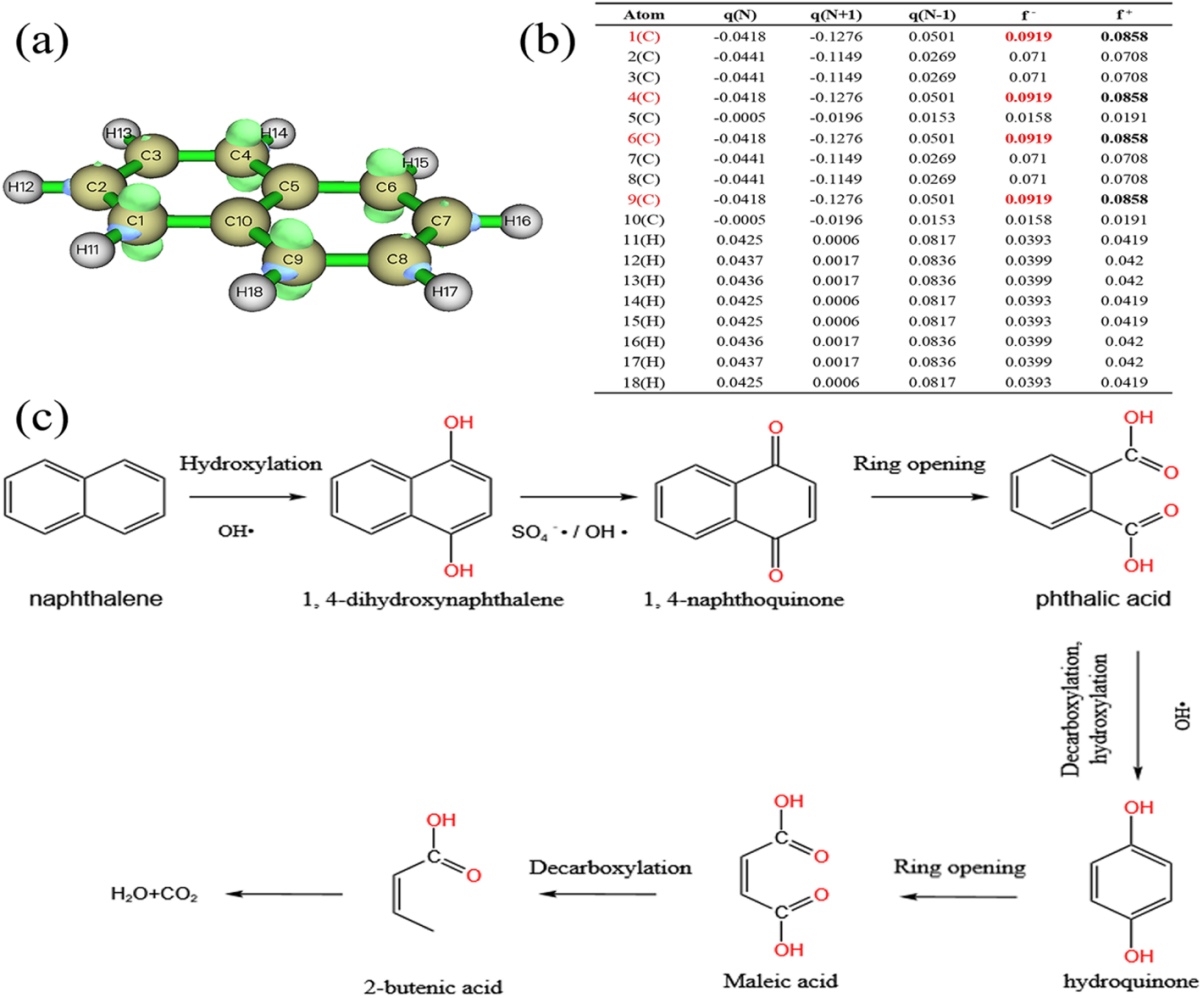
Chemical structure of NAP (a), Fukui index (b), possible degradation pathway of NAP (c).

As shown in Fig. 10b. In the calculation results, the index f^+^ represents the response to nucleophilic attacks, while the index f^−^ represents the response to electrophilic attacks.^88^ Atoms with larger index values are generally more reactive. For example, sites with higher f^−^ values in the molecule are more likely to be attacked by electrophilic free radicals SO_4_^−^˙ and OH˙,^89,90^ thus losing electrons. Therefore, C1, C4, C6 and C9 are easier to be attacked by SO_4_^−^˙ and OH˙ and take the lead in reaction. Studies^65,91^ have shown that aromatics mainly undergo hydroxylation, ring opening and decarboxylation in oxidation systems. Combined with the above conclusions, the possible degradation pathways of naphthalene were expounded, as shown in Fig. 10c.

## 4. Conclusions

A new type of magnetic activator was successfully prepared in this study. This activator exhibits excellent persulfate activation properties and can degrade naphthalene in wide pH range and coexistence of organic matter, anions. Radical quenching experiments showed that SO_4_^−^˙ and OH˙ were the main reasons for the removal of NAP. The possible degradation path of NAP was elucidated by DFT calculation. The prepared activator has good magnetic recovery performance and cycle stability. The use of magnetic recovery reduces the secondary pollution of the activator to water. Therefore, this magnetic porous activator provides a great potential for the removal of naphthalene and other pollutants in the environment.

## Author contributions

Shuaijie Gu conceived, designed and performed the experiments. Jingying Cui and Fangqin Liu analysed data and calculate. Shuaijie Gu and Jinyang Chen prepared the manuscript.

## Conflicts of interest

The authors indicate there are no conflicts to declare.

## Supplementary Material
